# Differential Diagnosis of Gastric Ulcer and Cancer

**Published:** 1881-04

**Authors:** 


Differential Diagnosis of Gastric Ulcer and Cancer.—Trousseau says on this subject, "Should the supposed cancerous tumor not be accessible to investigation, as in the case to which I have just alluded, there remains a valu ble diagnostic sign which I must indicate to you. This sign, to which, over fifteen years ago, I first called the attention of the profession, consists in the appearance of a *venous thrombosis*. When you are in doubt as to the nature of a gastric disorder, and are hesitating between a chronic gastritis, a simple ulcer, and a cancer, a *phlegmasia alba dolens* of the lower or the upper extremity will put an end to your indecision, and it will be allowable for you to assert positively the existence of a cancer.—*Pacific Medical and Surgical Journal.*

